# Correlation between CT findings and outcomes in 46 patients with coronavirus disease 2019

**DOI:** 10.1038/s41598-020-79183-4

**Published:** 2021-01-13

**Authors:** Qiang Lei, Guangming Li, Xiaofen Ma, Junzhang Tian, Yun fan Wu, Hui Chen, Wen Xu, Cheng Li, Guihua Jiang

**Affiliations:** 1 Department of Medical Imaging, Guangdong Traditional Medical and Sports Injury Rehabilitation Research Institute, Guangdong Second Provincial General Hospital, Haizhu District, Shiliugang Rd, Guangzhou, 510317 People’s Republic of China; 2 Department of Medical Imaging, Fancheng District, Xiangyang Central Hospital, Affiliated Hospital of Hubei University of Arts and Science, Zhongyuan RD, Xiangyang, 441003 People’s Republic of China

**Keywords:** Diseases, Health care

## Abstract

The aim of this study was to analyze initial chest computed tomography (CT) findings in COVID-19 pneumonia and identify features associated with poor prognosis. Patients with RT-PCR-confirmed COVID-19 infection were assigned to recovery group if they made a full recovery and to death group if they died within 2 months of hospitalization. Chest CT examinations for ground-glass opacity, crazy-paving pattern, consolidation, and fibrosis were scored by two reviewers. The total CT score comprised the sum of lung involvement (5 lobes, scores 1–5 for each lobe, range; 0, none; 25, maximum). 40 patients who recovered from COVID-19 and six patients who died were enrolled. The initial chest CTs showed 27 (58.7%) patients had ground-glass opacity, 19 (41.3%) had ground glass and consolidation, and 35 (76.1%) patients had crazy-paving pattern. None of the patients who died had fibrosis in contrast to six (15%) patients who recovered from COVID-19. Most patients had subpleural lesions (89.0%) as well as bilateral (87.0%) and lower (93.0%) lung lobe involvement. Diffuse lesions were present in four (67%) patients who succumbed to coronavirus but only one (2.5%) patient who recovered (p < 0.001). In the death group of patients, the total CT score was higher than that of the recovery group (p = 0.005). Patients in the death group had lower lymphocyte count and higher C-reactive protein than those in the recovery group (p = 0.011 and p = 0.041, respectively). A high CT score and diffuse distribution of lung lesions in COVID-19 are indicative of disease severity and short-term mortality.

## Introduction

Coronavirus Disease 2019 (COVID-19) has become a public health threat^1,2^. According to the World Health Organization, there have been 3,175,207 COVID-19 cases and 224,172 deaths with a mortality rate of 7.1% as of May 1, 2020^3^. The dominant symptoms include fever and respiratory discomfort. Gastrointestinal symptoms are rare, suggesting a difference in viral tropism compared with seasonal influenza, SARS-CoV, and MERS-CoV^4^.

Computed tomography (CT) findings at baseline and short-term CT follow-up have been published^5–9^. Typical CT imaging of COVID-19 include bilateral, peripheral, and basal predominant ground-glass opacity, consolidation, or both. Serial chest CT during recovery from COVID-19 shows four stages of lung abnormalities manifested as (1) ground-glass opacity with the development of (2) crazy-paving pattern and (3) increased consolidation (i.e., more extensive lung involvement), and (4) slow resolution. However, the severity of lung damage evaluated by CT and its differences between patients who recovered and those who expired have not been well studied. There is insufficient time from symptom onset to final outcome; therefore, it is essential to find early predictors of clinical outcomes.

Here, we describe in detail the distribution or the extent of lung abnormalities in COVID-19 patients upon admission and analyze its association with final outcomes. The purpose of this study was to identify the features and draw specific patterns of lung abnormalities on admission to facilitate better clinical decisions, timely therapeutic strategy, and reduce mortality.

## Material and methods

### Study design and patients

This retrospective study was approved, and written informed consent was waived by the medical ethics committee of Guangdong Second Provincial General Hospital (protocol number 352072440105210132). We retrospectively reviewed patients diagnosed with COVID-19 who were admitted to Guangdong Second Provincial General Hospital or Xiangyang Central Hospital from 1 January 2020 to 20 February 2020. The patients recovered or died within 2 months of hospitalization, and the interval between the first or last CT scan and reverse transcription polymerase chain reaction (RT-PCR) was less than 3 days. Patients with COVID-19 confirmed by RT-PCR testing were enrolled, and both chest CT scan and RT-PCR testing were conducted within an interval of three or fewer days.

The exclusion criteria were (1) patients who refused chest CT, (2) severe motion artifacts on chest CT, and (3) patients whose outcomes were not available within 2 months of hospitalization. Basic clinical characteristics and initial CT features were compared between two groups on the basis of clinical outcomes: the death group and the recovery group.

### Diagnostic and recovery criteria of COVID-19

According to the preliminary diagnosis and treatment protocols from the National Health Commission of the People’s Republic of China, the final diagnosis for COVID-19 was determined by real-time fluorescence polymerase chain reaction from oropharyngeal swabs of suspected patients. Patients with confirmed COVID-19 were hospitalized and isolated for treatment. The recovery criteria were as follows: (1) no fever for more than 3 days, (2) respiratory symptoms significantly improved, (3) improvement in the radiological abnormalities on chest CT, and (4) at least two negative COVID-19 nucleic acid detection within an interval of 1 day.

### Chest CT protocols

All images were obtained on Optima 660 (GE, USA) with patients in the supine position. The main scanning parameters were as follows: tube voltage = 120 kV, automatic tube current modulation (30–70 mAs), pitch = 0.99–1.22 mm, matrix = 512 × 512, slice thickness = 10 mm, field of view = 350 mm × 350 mm. All images were then reconstructed with a slice thickness of 0.625–1.250 mm with the same increment^10^.

### CT image analysis

All chest CT images were reviewed by two radiologists (Q.L. and G.M.L with 5 and 15 years of experience in interpreting chest CT imaging, respectively) including main CT features and lesion distribution. The total CT score was determined by consensus. Referring to international standard nomenclature defined by the Fleischner Society glossary^11^ and peer-reviewed literature on viral pneumonia^12,13^, the main CT features were described using terms including ground-glass opacity (GGO), crazy-paving pattern, consolidation, and fibrosis. Reticulation with associated architectural distortion and mild traction bronchiectasis were considered to be related to fibrosis.

The lung involvement of all these abnormalities was quantitatively estimated according to the area involved. Each lung lobe was visually scored from 0 to 5; 0, no involvement; 1, < 5% involvement; 2, 25% involvement; 3, 26–49% involvement; 4, 50–75% involvement; 5, > 75% involvement. The total CT score was the sum of the individual lobar scores and ranged from 0 (no involvement) to 25 (maximum involvement). The distribution of pulmonary abnormalities was classified as predominantly subpleural (mainly the peripheral one-third of the lung involved), diffuse (continuous involvement without respect to lung segments), or random^5^.

### Statistical analysis

Statistical analyses were performed using IBM SPSS Statistics Software (version 23.0; IBM, Chicago, IL, USA). Normally distributed data were presented as mean (SD), non-normally distributed data as median (IQR), and categorical data as the percentage of the total unless otherwise specified. The comparisons of non-paired data were evaluated using the Student *t* test, Mann–Whitney *U* test, or Chi-squared test^5^. A p-value of < 0.05 was defined as statistically significant.

### Ethics approval

All procedures performed in studies involving human participants were in accordance with the ethical standards of the institutional and/or national research committee and with the 1964 Helsinki declaration and its later amendments or comparable ethical standards.

### Informed consent

This retrospective study was approved by the medical ethical committee of Guangdong Second Provincial General Hospital, and informed written consent was waived.

## Results

### Patient characteristics

There were 46 patients (26 males and 20 females) included in the study. The average age was 50 (17) years (range 9–87). 40 patients were assigned to recovery group and six patients to death group. Patients who succumbed to COVID-19 pneumonia were older than those who recovered (p = 0.001). Patients with hypertension were more likely to die than those without the previous history of d hypertension (p = 0.002; Table 1).

**Table 1:** Comparison of final outcome according to clinical characteristics.

Clinical features	Outcome		P
	Recovery (n = 40)	Non-survivors (n = 6)	
**Sex**			0.328
Male	21 (52.5)	5 (83.3)	
Female	19 (47.5)	1 (16.7)	
**Age (years)**			0.002
≤ 60	34 (85.0)	1 (16.7)	
> 60	6 (15.0)	5 (83.3)	
**Fever**			0.169
Yes	36 (90.0)	4 (66.7)	
No	4 (10.0)	2 (33.3)	
**Cough**			0.624
Yes	21 (52.5)	3 (50.0)	
No	19 (47.5)	3 (50.0)	
**Hypertension**			0.002
Yes	6 (15.0)	5 (83.3)	
No	34 (85.0)	1 (16.7)	
**Diabetes**			0.120
Yes	3 (7.5)	2 (33.3)	
No	37 (92.5)	4 (66.7)	
**Laboratory**			
Leukocyte (× 10^9^ cells/L)	5.3 (2.5)	4.7 (3.1)	0.683
Lymphocyte (× 10^9^ cells/L)	2.3 (2.4)	1.3 (0.8)	0.011
C-reactive protein (mg/L)	17.8 (46.0)	125.5 (174.8)	0.041

Continuous data presented as median (IQR) and categorical data as n (%).

The most prevalent presenting symptoms include fever (22, 48.0%), cough (6, 13.0%), or both (18, 39.0%). These patients underwent initial CT scans from the symptom onset with a mean interval of 5 (3) days (range 1–15 days). A total of 86 chest CT scans (46 initial CT and 40 discharge CT re-examination) were performed. After initial symptom onset, 40 patients recovered with a mean hospitalized period of 23 (9) days (range 9–42), and six patients died after a mean hospitalized period of 16 (8) days (range 8–25).

### Chest CT evaluation

**Figure 1:**
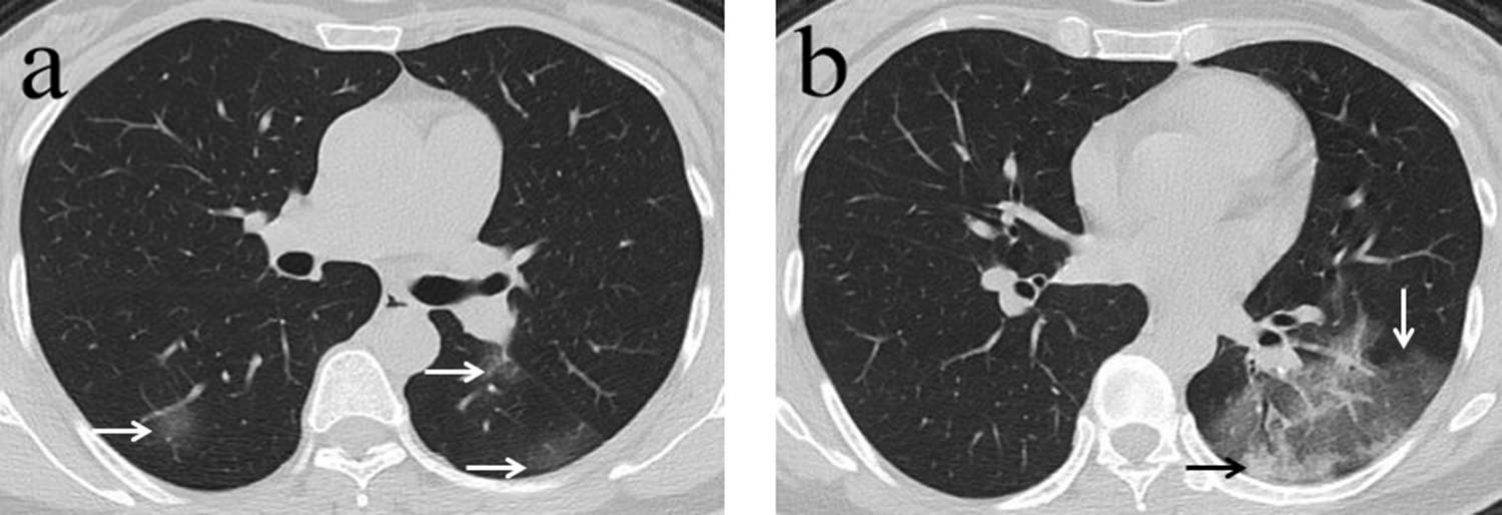
A 45-year-old woman with history of recent travel to Wuhan presented with fever for 1 day. (**a**,**b**) Non-contrast enhanced chest computed tomography (CT) showed multiple peripheral patchy ground glass opacities in both lower lobes (white arrows). CT scan also demonstrated consolidation in the left lower lobe (black arrow). CT involvement score was 5.

**Figure 2:**
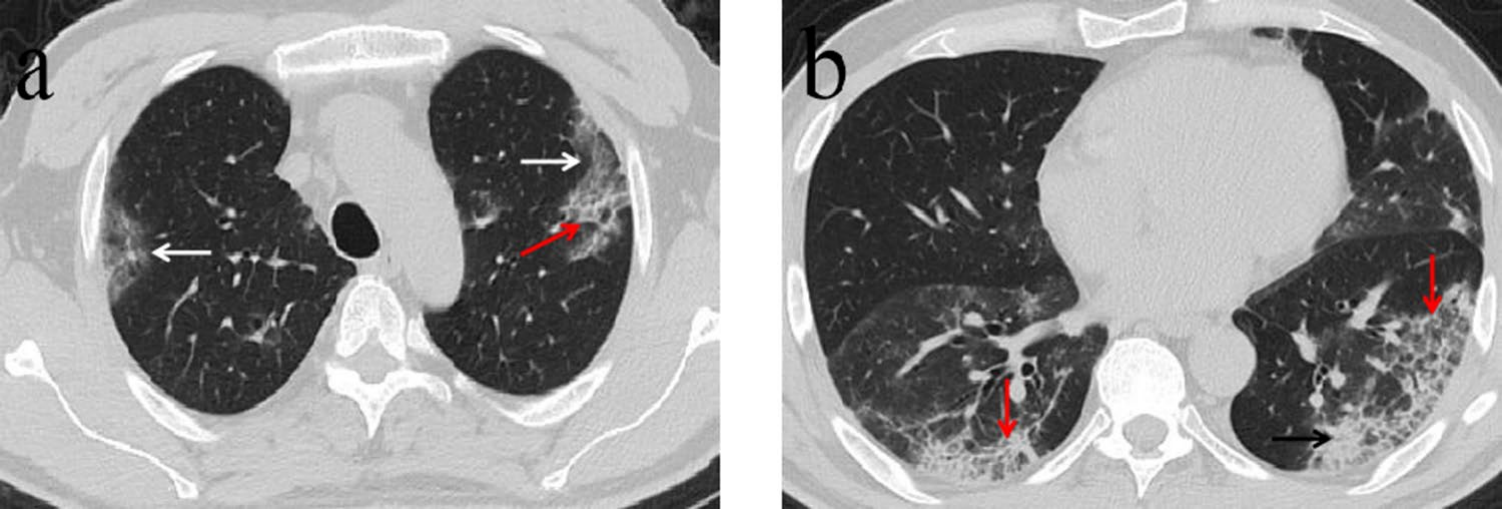
A 57-year-old man with a history of recent travel to Wuhan presented with fever for 10 days. (**a**,**b**) Non-contrast enhanced chest CT showed multiple peripheral patchy ground glass opacities (white arrows) and crazy-paving pattern (red arrow) in both lower lobes. CT scan also demonstrated consolidation in the left lower lobe (black arrow). CT involvement score was 11.

Ground-glass opacity (GGO), crazy-paving pattern (GGO with superimposed inter- and intralobular septal thickening), and consolidation were the main CT findings in early-stage COVID-19 pneumonia (Figs. 1, 2). Of all patients examined, 27 (58.7%) had ground-glass opacity, 19 (41.3%) had ground-glass and consolidation in combination, and 35 (76.1%) patients had crazy-paving pattern. Fibrosis was observed in six (6/40, 15.0%) patients who recovered, and none of the six patients died with COVID-19. Cavity, mediastinal lymphadenopathy, and pleural effusion were not observed. The number of lung lobes involved in those who died of COVID-19 showed no significant differences versus those who recovered (p = 0.096). In those patients who died, the total CT score was greater than those who recovered (p = 0.005). Finally, subpleural lesions (41/46, 89.0%) were more common than central or diffuse lung lesions (5/46, 11.0%). Of the patients who died, four (4/6, 67.0%) had diffuse lesions while only one (1/40, 2.5%) patient who recovered had diffuse lesions. Patients with diffuse lung lesions were more likely to die than those with subpleural lesions (4/5, 80.0% vs 2/41, 4.8%, p < 0.001; Table 2). Most patients had bilateral (40/46, 87.0%) and lower (43/46, 93.0%) lung lobe involvement.

**Table 2:** Comparison of final outcome according to CT findings.

CT findings	Outcome		P
	Recovery (n = 40)	Non-survivors (n = 6)	
**Ground-glass opacity**			0.680
Yes	24 (60.0)	3 (50.0)	
No	16 (40.0)	3 (50.0)	
**Ground-glass opacity plus consolidation**			0.680
Yes	16 (40.0)	3 (50.0)	
No	24 (60.0)	3 (50.0)	
**Crazy paving**			1.000
Yes	30 (75.0)	5 (83.3)	
No	10 (25.0)	1 (16.7)	
**Distribution**			< 0.001
Subpleural	39 (97.5)	2 (33.3)	
Diffuse	1 (2.5)	4 (66.7)	
Number of lung lobes involved	4.0 (2.0)	5.0 (0.7)	0.096
CT score	6.0 (5.0)	20.5 (14.0)	0.005

Continuous data presented as median (IQR), categorical data as n (%), *CT *computed tomography.

## Discussion

The main CT findings of COVID-19 consist of ground-glass opacity, consolidation, and crazy-paving pattern, which were similar to those described by several groups of investigators^14,15^. Our study highlights the clinical implication of initial CT findings as a prognostic indicator in patients with COVID-19. Upon comparing those who recovered with those who did not, neither ground-glass opacity, consolidation, nor crazy-paving pattern were associated with final outcomes. However, fibrosis was observed in six who recovered and none who died. We speculate that fibrotic changes may have resulted from organizing pneumonia patterns of lung injury with COVID-19. Jae et al.^1,6^ found the remaining opacity of MERS in the intubation group and that fibrosis was a sequela on follow-up chest radiographies. Similar to other acute lung injuries, fibrosis is thought to be the result of resolution^13,17^. Fibrosis was in the early stages, assuming all of the patients were in an early stage of infection and underwent initial CT scans from symptom onset with a mean interval of 5 ± 3 days. This may be associated with good prognosis in early chest CT examinations. In our patients, pleural effusion was not present in those with COVID-19. Karuna et al.^18^ reported that the early appearance of pleural effusion was a predictor of short-term mortality in patients with MERS; however, this relationship was not demonstrated in our patients with COVID-19.

A high CT lung score was a sign of poor prognosis and was associated with short-term mortality. Patients who died had higher CT lung scores versus those who recovered in our study, indicating the rapid development of the disease process. The largest area of the lung was occupied by pneumonia. Similar to our study, Zhao et al.^19^ reported that the CT involvement score can help evaluate the severity and extent of COVID-19. The area of the affected lung lobes in expired patients was greater than those who recovered. The predominant peripheral distribution of abnormalities was observed in our study; however, four patients who died and one who recovered showed diffuse distribution of lung lesions. Patients with diffuse lung lesions were more likely to die than those with subpleural lesions. These factors are consistent with diffuse alveolar damage patterns that may cause worse outcomes for patients. Xu et al.^20^ also reported a COVID-19 patient death with diffuse lung involvement on X-ray images and pathological findings of pulmonary edema and clear film formation on lung biopsy samples. These findings indicate acute respiratory distress syndrome (ARDS). Our results suggest that high CT scores coupled with diffuse distribution of lung lesions on admission were responsible for ensuing ARDS and a poor prognosis in most patients.

Patients who died from COVID-19 had a higher C-reactive protein and lower lymphocytic count than the recovery group. Lymphocytes were reduced in the group that died, reflecting a deficiency of adaptive immune response. Lymphopenia and high plasma levels of C-reactive protein may be related to cytokine storm in patients with COVID-19 as postulated by a previous report^1^. In our study, older subjects, and those with hypertension were statistically associated with mortality; however, we did not see a statistically significant difference between the two groups in terms of sex and diabetics. These factors were congruent with the MuLBSTA score^21^ for predicting mortality in viral pneumonia and may have played a role in the death of patients with COVID-19.

Our study has several limitations. The sample size especially in the death group was small; negative results and infections with other viruses were not included. Future studies should involve a large patient population. Also, the time of the initial CT after admission was not unified, and imaging changes in COVID-19 are rapid.

## Conclusion

In conclusion, a high CT score and diffuse distribution of lung lesions in COVID-19 are indicative of a poor prognosis and short-term mortality.

## Data Availability

All the materials and data in studies are available.
